# Parental chromosomal heteromorphisms are not associated with an increased risk of embryo aneuploidy

**DOI:** 10.5935/1518-0557.20210011

**Published:** 2021

**Authors:** Carlos Hernandez-Nieto, Sonia Gayete-Lafuente, Tamar Alkon-Meadows, Joseph Lee, Martha Luna-Rojas, Tanmoy Mukherjee, Alan B Copperman, Benjamin Sandler

**Affiliations:** 1 Reproductive Medicine Associates of New York, New York, USA; 2 Obstetrics, Gynecology and Reproductive Science, Icahn School of Medicine at Mount Sinai, New York, USA

**Keywords:** Preimplantation genetic testing (PGT), embryo aneuploidy, chromosomal heteromorphisms, heterochromatic variants, *in vitro* fertilization

## Abstract

**Objective:**

Although chromosomal heteromorphisms are commonly found in the general population, some researchers have suggested a correlation with higher rates of embryo aneuploidy. This study aimed to assess the rates of embryo aneuploidy in couples who carry a chromosome heteromorphism.

**Methods:**

The study included couples who had G-banding karyotype testing and underwent an IVF/PGT-A cycle between January 2012 and March 2018. The participants were classified by couple karyotype: Group A: ≥1 patient reported to be a heterochromatic variant carrier; Group B: both partners reported to be “normal”. We assessed the rates of aneuploidy among the groups. We ran a multivariate regression analysis to assess the relationship between heterochromatic variants and the rates of embryo aneuploidy.

**Results:**

Of the 946 couples analyzed, 48 (5.0%) reported being a carrier of ≥1 heterochromatic variant. We had 869 IVF/PGT-A cycles included in the analysis (Group A: n=48; Group B: n=82). There were no significant differences in embryo ploidy rates among the groups. The heterochromatic chromosome variant was not associated with increased likelihoods of aneuploidy (OR=1.04, CI:95% 0.85- 1.07; *p*=0.46). Finally, the gender of the heterochromatic variant carrier had no association with increased likelihood of aneuploidy (OR 1.02, CI 95% 0.81-1.28, *p*=0.82).

**Conclusions:**

Our study showed no association between parental heterochromatic chromosome variants and subsequent embryo aneuploidy rates. Ploidy rates do not appear to be negatively associated with couples when at least one patient is reported to be a carrier of a heterochromatic variant on the karyotype.

## INTRODUCTION

Heterochromatic Chromosomal Variants or Chromosomal heteromorphisms (CH) are quantitative or positional alterations in constitutive DNA heterochromatin, occurring in the centromeric region of chromosomes 1, 9, 16, Y, and short arms of acrocentric chromosomes. These alterations or variants, named secondary constriction regions (qh), can include different length patterns for heterochromatin blocks (marked as qh+ or qh-), or even the inversion of an entire block ^(Mierla & Stoian, 2012; Šípek et al., 2014)^. CH were considered to be normal familiar variants due to their frequency in the general population, after karyotyping staining techniques ^(Dong *et al*., 2013; Bhasin, 2005; Wyandt & Tonk, 2012)^. Today, these heterochromatic regions are known to sustain non-coding repetitive sequences of satellite DNA, which regulates and modulates gene expression under different cellular function conditions. Additionally, these regions contribute to proper chromosome segregation by binding sister centromeres and assembling the kinetochore during meiosis ^(Skaletsky *et al*., 2003)^. Centromere and kinetochore dynamics are essential for correcting or avoiding abnormal chromosome segregation during gamete and/or embryo development processes ^(Yakin *et al*., 2005)^.

The true clinical significance of carrying these heteromorphisms and their impact on human gametes and post-fertilization embryo development has not yet been fully elucidated. Several studies have reported a higher incidence of these variants in couples, affecting both male and females suffering from infertility, recurrent pregnancy loss, or reproductive failure ^(Šípek et al., 2014; Morales *et al*., 2016; Düzcan *et al*., 2003; Madon *et al*., 2005; Iyer *et al*., 2007; Sahin *et al*., 2008; Akbaş *et al*., 2012; Delhanty *et al*., 1997; Mantzouratou *et al*., 2007)^. However, the relationship between chromosomal heteromorphisms and embryonic chromosomal composition in ART treatments remains predominantly speculative. Thus, the objective of our study is to evaluate whether couples undergoing ART treatments who carry any CH are at risk of producing embryos with a higher incidence of aneuploidy.

## MATERIALS AND METHODS

### Design: Retrospective cohort analysis

The study included couples with G-banding karyotype testing on peripheral blood lymphocytes who underwent an IVF/ICSI cycle, with preimplantation genetic testing for aneuploidy (PGT-A), from 2012 to 2018. The couples were separated into groups based on their karyotype results (Group A: ≥1 patient reported to be a heterochromatic variant carrier; Group B: both partners reported to be “cytogenetically normal”). Indications for karyotype testing and PGT-A included: patients with a history of pregnancy losses, aneuploidy, prior stillbirth, poor embryonic quality, and severe male factor infertility. Couples in which one or both partners were found to be carriers of any balanced translocation, chromosomal inversions, mosaicisms, or known pathogenic polymorphisms were excluded from the analysis. Additionally, patients diagnosed with severe male factor and those who required testicular sperm extraction and/or ovum donation were excluded from the study.

### IVF and laboratory procedures

The patients underwent ovarian stimulation for IVF, all stimulation protocols, laboratory procedures, and embryo morphology grading specifics have been previously described elsewhere ^(Hernandez-Nieto *et al*., 2019)^. All oocytes were inseminated with ICSI and underwent extended culture to the blastocyst stage of development. Trophectoderm (TE) biopsies were submitted to chromosome copy number analysis through quantitative real-time PCR (qPCR), and/or next generation sequencing (NGS) based analysis. The biopsy results were interpreted as euploid, aneuploid, or inconclusive result, as described previously ^(Hernandez-Nieto *et al*., 2017)^, mosaic embryos were catalogued as aneuploid. All inconclusive result embryos that underwent a second TE biopsy were excluded from the analysis.

Euploidy rate was defined as the number of euploid embryos over the number of embryos biopsied.

### Statistical methods

The statistical analysis was performed using SAS version 9.4 (SAS institute Inc., Cary, NC, USA). The descriptive data was compared by T-test, ꭓi2 test and Mann-Whitney U tests when appropriate. All results were expressed as percentages, means and Standard Deviations (SD). Adjusted odds ratios (OR) with 95% CI were calculated using a multivariate logistic regression analysis to assess the effect of a CH on any of the patients and the odds of increased embryo aneuploidy.

The logistic regression models were fitted with generalized estimating equations (GEE) to account for patients who underwent multiple cycles. All variables that showed significance and were thought to be clinically relevant were included as covariates in the model. All p-values are two-sided with a clinical significance level of *p*<0.05.

## RESULTS

869 couples underwent karyotype testing, of those, 48 (5.0%) were found to be a carrier of a CH. There were no couples in which both partners carried a CH in the study. The most common CH types found were non-pathogenic pericentric inversions on chromosome 9 (15/48, 31.2%) and block variants (qh) on chromosome Y (11/48, 22.9%). CH were more common in male partners (32/48, 66.6%) than in females (16/48; 33.3%). All the cases had heterozygosity in the CH carrier. A total of 869 IVF/PGT-A cycles were included in the analysis (Group A “carriers”: n=48; Group B “normal karyotypes”: n=821). Of those cases, 4,017 trophectoderm biopsies were analyzed (Group A: n=264; Group B: 3753). Demographic, stimulation parameters and embryological variables were comparable among cohorts (Table 1).

**Table 1:** Comparison analysis of demographic, oocyte stimulation, embryological and ploidy variables of the populations analyzed.

Variable	Variant carrier n=48 cycles	Normal Karyotype n=821 cycles	*p*-value
Age (years)	36.22±4.22	36.37±4.16	0.81
Body Mass Index (Kg/m^2^)	22.82±3.61	28.42±143.12	0.27
Day of ovulation trigger	12.06±1.42	12.91±13.88	0.11
Gonadotropin Cumulative Dose (IU)	3988.47±1562.28	3769.04±1337.67	0.27
Estradiol at trigger (pg/mL)	2321.91±880.83	2447.39±1170.89	0.35
Progesterone at trigger (ng/mL)	0.86±0.42	0.94±0.54	0.27
Baseline FSH (IU/mL)	6.44±3.78	6.09±3.37	0.57
Anti-Mullerian Hormone (ng/mL)	3.70±4.65	3.28±3.45	0.52
Antral follicle count (n)	12.23±6.68	12.33±5.97	0.91
Oocytes Retrieved (n)	15.85±9.56	15.77±8.49	0.95
Mature (MII) oocytes (n)	12.02±8.11	11.78±7.07	0.81
2PN embryos (n)	9.35±6.89	9.54±6.03	0.83
Blastocysts biopsied (n)	5.62±4.67	4.69±3.67	0.18
Aneuploid embryos (n)	2.70±2.60	2.08±1.89	0.11
Euploid embryos (n)	2.57±2.73	2.44±2.67	0.73
Inconclusive results (n)	0.33±0.93	0.17±0.63	0.24

Note: Data presented as means±standard deviation, unless stated otherwise. Statistical significance, *p*<0.05.

Oocyte maturity rates between CH carriers and non-carriers were comparable (Group A: 77.40%; Group: 80.30%; *p*=0.91); similarly, fertilization rates (Group A: 77.80%; Group B: 80.30%; *p*=0.61); blastulation rates (Group A: 71.20%; Group B: 64.60%; *p*=0.19) and mean number of biopsied/cryopreserved blastocysts per cycle (Group A: 82.50%; Group B: 74.10%, *p*=0.21). In a multivariate regression analysis adjusted for female and male patients’ age, BMI, and AMH levels, there was no association with the presence of a CH variant and lower odds of blastulation (OR 1.85, CI 95% 0.56-6.10). There were no significant differences in embryo euploidy rates (Group A: 45%; Group B: 52%; *p*=0.61), aneuploidy rates (A: 48%; B: 44%; *p*=0.98) or inconclusive report rates (A: 7%; B: 3.7%; *p*=0.32) (Table 2).

**Table 2:** Outcomes of oocytes retrieved and ploidy rates of the populations analyzed.

Variable outcome	Variant Carrier		Normal Karyotype		*p*-value
	N	%	N	%	
Oocytes retrieved	745		12633		0.95
Mature oocytes rate	577/745	77.40%	9750/12633	77.10%	0.95
Fertilization rate	449/577	77.80%	7835/9750	80.30%	0.61
Blastulation rate	320/449	71.20%	5064/7835	64.60%	0.19
Biopsied blastocyst rate	264/320	82.50%	3753/5064	74.10%	0.21
Euploid embryos rate	121/264	45%	1951/3753	52%	0.61
Aneuploid embryos rate	127/264	48%	1660/ 3753	44%	0.98
Inconclusive reports rate	20/264	7%	142/3753	3.70%	0.32

Note: Data presented as percentages, unless stated otherwise. Statistical significance, *p*<0.05.

In a multivariate regression analysis adjusted for female and male patients age, body mass index, AMH, and day when embryo biopsy was performed. There was no association with the presence of a CH variant and increased odds of aneuploidy (OR= 1.04, CI95% 0.92 - 1.18). A sub-analysis adjusted for gender and CH carrier status found no association with the presence of a CH and increased odds of embryo aneuploidy when comparing male versus female carriers (OR 1.02, CI 95% 0.81-1.28).

## DISCUSSION

As heterochromatin plays an essential role in meiosis, the presence of CH has been theorized to impair the formation of functional gametes. Consequently, patients who are CH carriers might theoretically be more susceptible to experiencing an increased incidence of embryonic aneuploidy and impaired reproductive outcome ^(Morales *et al*., 2016)^. Our study suggests that couples carrying CH are not at greater risk of experiencing increased rates of embryo aneuploidy when compared to non-carriers. The most common CH found in this study involved pericentric inversions of chromosome 9 (31.2%); in second, variants in detectable regions of the Y chromosome (22.9%). According to the literature, chromosome 9 pericentric inversions are the most frequent CH in infertile patients, but also in the general population, followed by 9qh+ and 9qh- variants ^(Ferguson-Smith, 1974; Verma *et al*., 1978; Humphray *et al*., 2004; Codina-Pascual *et al*., 2006; Collodel *et al*., 2006; Penna Videaú *et al*., 2001; Nagvenkar *et al*., 2005; Ferguson *et al*., 2007; 2009; Sarrate *et al*., 2014; García-Peiró *et al*., 2011a; 2011b)^.

Although some studies have addressed the potential associations of CH on fertility treatments, our study found no differences in ovarian stimulation parameters, number of oocytes retrieved and fertilization rates during IVF/ICSI cycles. Conversely, one study by ^Guo *et al.* (2012)^ found lower fertilization rates in CH-carrying men with severe oligozoospermia, compared with non-carriers also with severe oligozoospermia. Thus, suggesting that CH might have detrimental effects on spermatogenesis and a negative impact on IVF outcomes ^(Guo *et al*., 2012)^. Additionally, ^Liang *et al*. (2014)^ reported lower fertilization rates in couples in which only the male carried the CH, compared with couples with only a female CH-carrier and infertile couples with normal karyotypes. Notably, these aforementioned studies included cohorts of mixed conventional IVF and ICSI cases. In our study, all cases underwent ICSI as insemination method, and our results showed similar fertilization rates among patients who were CH carriers and non-carriers (80.3% *vs*. 77.8%, *p*=0.61).

Our data suggests that embryonic blastulation, embryonic morphological quality and euploidy rates are not significantly associated with the presence or absences of CH on couple’s karyotypes. A study by ^Xu *et al*. (2016)^ reported that CH in either male or female carriers seemed to have adverse effects on IVF/ICSI-ET outcomes. That study suggested that CH in male carriers affected IVF outcomes by decreasing the rates of fertilization, number of available cleavage stage embryos, good quality embryos and clinical outcomes after the transfer of these embryos. In addition, the presence of CH in female carriers affected outcomes only by lowering the embryo cleavage rate. To our knowledge, this deleterious effect has yet to be reported for extended culture of the embryo to the blastocyst stage. Our study is the first to demonstrate that blastulation rates (64.4% *vs*. 71.2%, *p*=0.19) and total number of cryopreserved and biopsied blastocysts (74.1% *vs*. 82.5%, *p*=0.21) were not adversely affected by CH carrier status, regardless of paternal or maternal contribution.

A retrospective analysis by ^Morales *et al*. (2016)^, examined the relationship between CH, infertility and aneuploidy in sperm cells and embryos. Apart from observing a high prevalence of CH among infertile patients when compared to a control group, (19.4% *vs*. 13.4%; *p*<0.01), they reported major rates of sperm aneuploidy in CH carriers (37.7% *vs*. 16.3%; *p*<0.01). Further, they found an increased rate of embryo aneuploidy rates in female carriers than in non-carrier oocyte recipients (102 embryos from CH carriers with a reported aneuploidy rate of 50.0% compared with 199 embryos of a control group yielding a 27.6% aneuploidy rate (*p*<0.001). Conversely, after analyzing 4,017 blastocysts, our study’s results showed no significant differences in embryo ploidy rates among groups. Furthermore, after utilizing an adjusted analysis controlling for patient’s age and other potential confounders, there was no association with the presence of a CH variant and increased odds of embryo aneuploidy. Additionally, there was no association with the odds of aneuploidy after performing a sub-analysis that adjusted for the sex of the CH carrier. One of the main differences from our study compared to that from ^Morales *et al*. (2016)^, was our use of the most contemporary technology available for genetic assessment. While they examined the embryos by a-CGH, we analyzed embryonic chromosomal composition with more modern clinically validated platforms including NGS ^(Friedenthal *et al*., 2018; Lee *et al*., 2015)^.

Despite our best effort to avoid biases on the study, this analysis still has limitations, as its retrospective nature increases the likelihood of a selection bias. One limitation to consider is the lack of standardization for karyotyping indications in patients and couples who undergo ART. The cytogenetically karyotyped individuals in our study included patients with refractory infertility or prior history of poor IVF outcomes or embryonic quality, or couples with a history of recurrent losses and implantation failure. Also, not all the karyotyping was performed in a same laboratory. Some commercial laboratories do not consider CH as normal variants and exclude from their report, differences in reporting might underestimate the CH prevalence in the general population. Finally, we did not include the pregnancy or perinatal outcomes of the studied embryos in our study, as these outcomes were beyond the scope of our analysis.

One of the strengths of our study is that all the clinical data analyzed came from a single, high volume center experienced in blastocyst trophectoderm biopsy. Our study includes one of the largest cohort of embryos chromosomally screened in couples carrying a CH, also, we compared the embryo aneuploidy rate from all infertile patients with multiple infertility diagnoses (CH carrier vs non-carrier). Moreover, we excluded clinical diagnoses associated with a significant increase in aneuploid embryos during ART such as carriers of balanced translocations, inversions, mosaicisms, or known pathogenic polymorphisms ^(Petracchi *et al*., 2009; Warburton & Fraser, 1964; Hassold *et al*., 1980)^. Concordant to previous published studies, we excluded patients with severe male factor utilizing testicular sperm extraction, these group of patients had been previously reported to be associated with a higher frequency of Y chromosome variants compared to fertile men ^(Morales *et al*., 2016; Penna Videaú *et al*., 2001; Nagvenkar *et al*., 2005; Guo *et al*., 2012)^ and with an increased rate of sperm aneuploidy ^(Yakin *et al*., 2005; Morales *et al*., 2016; Ferguson *et al*., 2007; García-Peiró *et al*., 2011a; 2011b; Mozdarani *et al.*, 2007; Minocherhomji *et al*., 2009)^; both contributors to an increased risk of embryonic aneuploidy. Finally, our study utilized an adjusted multivariate analysis fitted with a GEE that accounted for patients who underwent multiple cycles and controlled for other potential cofounders (i.e. patients age, BMI, AMH, ovarian stimulation parameters, and embryological variables).

The relatively high incidence of CH observed in infertile patients demands the need to evaluate the potential associations with infertility and subfertility. The relationship between ‘normal’ CH and reproductive outcome remains highly contested; epigenetic, genetic, and chromosomal modifications have been associated with infertility and poor reproductive outcomes ^(Morales *et al*., 2016; Collodel *et al*., 2006; Penna Videaú *et al*., 2001; Nagvenkar *et al*., 2005; Ferguson *et al*., 2007; 2009; Sarrate *et al*., 2014; García-Peiró *et al*., 2011a; 2011b)^. However, the findings of our study showed that there is no association between parental CH and increased risk of embryonic aneuploidy. Ploidy rates do not appear to be negatively associated with couples when at least one patient is reported to be a carrier of a heterochromatic variant on karyotype. Further analysis with high-resolution genome karyomapping and haplotyping technology may unveil potential relationships between parental chromosomal variants and embryological chromosome segregation errors and give us further insight on their interaction with embryonic development.
